# Gene Polymorphisms of *TLR2* Subfamily and Bacterial Meningitis in Angolan Children

**DOI:** 10.3390/genes17030260

**Published:** 2026-02-25

**Authors:** Johanna Teräsjärvi, Elina Tenhu, Manuel Leite Cruzeiro, Okko Savonius, Emilie Rugemalira, Qiushui He, Tuula Pelkonen

**Affiliations:** 1 Infection and Immunity, Institute of Biomedicine, University of Turku, 20520 Turku, Finland; johter@utu.fi (J.T.); elinaptenhu@gmail.com (E.T.); 2 Hospital Pediátrico David Bernardino, Luanda Cx.P. 3067, Angola; mcruzeiro1@gmail.com (M.L.C.); tuulapelkonen@hotmail.com (T.P.); 3 Department of Paediatrics, Helsinki University Hospital, University of Helsinki, Stenbäckinkatu 9, 00290 Helsinki, Finlandemilie.rugemalira@helsinki.fi (E.R.); 4 New Children’s Hospital, Pediatric Research Center, 00290 Helsinki, Finland; 5 InFLAMES Research Flagship Center, University of Turku, 20520 Turku, Finland

**Keywords:** bacterial meningitis, pneumococcal meningitis, TLR2 subfamily, TLR2, TLR10, SNP, *Streptococcus pneumoniae*, children, Angola

## Abstract

Background/Objectives: Bacterial meningitis is a severe disease with a fatality rate of 5–50%. It is mainly caused by *Streptococcus pneumoniae* or *Neisseria meningitidis*, which can also cause simultaneous infections outside the central nervous system. Toll-like receptors (TLRs) have an important role in the innate immune system. The TLR2 subfamily comprises the four highly homologous members TLR1, TLR2, TLR6, and TLR10, which also have an important immunomodulatory role in infectious diseases. Methods: The study cohort consists of 190 bacterial meningitis patients aged 1 to 147 months from randomized clinical trials and 268 controls from Luanda, Angola. Polymorphisms of *TLR2* (rs111200466) and *TLR10* (rs10856837 and rs11096956) were determined using PCR-based methods and Sanger sequencing. The genotyping results were analyzed together with clinical data to determine whether gene polymorphisms of *TLR2* and *TLR10* are associated with susceptibility and outcome of bacterial meningitis in Angolan children. Results: At admission and during hospitalization, patients with pneumococcal meningitis carrying a variant (ins/del or del/del) of *TLR2* rs111200466 had a significantly lower risk of coexisting infections (OR 0.27; 95% CI 0.11–0.65; *p* = 0.004), particularly pneumonia (OR 0.18; 95% CI 0.06–0.49; *p* = 0.001). In addition, haplotype analysis demonstrated that a variant genotype of *TLR2* rs111200466 together with a wildtype of *TLR10* SNPs (rs10856837 and rs11096956) may protect against coexisting pneumonia (OR 0.2; 95% CI 0.06–0.6; *p* = 0.007). Conclusions: This study suggests an association between coexisting infection and genetic variation in *TLR2* and *TLR10* of bacterial meningitis in Angolan children.

## 1. Introduction

Bacterial meningitis (BM) is a severe disease in which the fatality rate varies from 5% to 50%, and neurological sequelae affect up to 50% of survivors. There are approximately two million cases of BM each year, although vaccination programs have reduced its occurrence. BM is mostly caused by *Streptococcus pneumoniae*, *Neisseria meningitidis*, group B *Streptococcus* and *Haemophilus influenzae*. These pathogens are common in the nasopharynx (NP) without symptoms, and transmission can occur through respiratory droplets or saliva [1]. BM commonly occurs together with other infections outside the central nervous system, including pneumonia, bacteremia, sinusitis, and otitis media [2,3].

Toll-like receptors (TLRs) are a group of pattern recognition receptors (PRRs), which have an important role in pathogen recognition and the induction of innate immune responses [4]. In humans, ten TLRs, from TLR1 to TLR10, have been identified. They are known to interact with different ligands through the extracellular domain [4]. The TLR2 subfamily comprises four highly homologous members: TLR1, TLR2, TLR6, and TLR10 [5]. TLR2 functions either as a homodimer or in pairwise combinations with TLR1 or TLR [6], playing an essential role in recognizing bacterial lipoproteins and lipopeptides [5,6]. TLR10 forms homodimers as well as heterodimers with TLR1 and TLR2, although its ligand remains unknown [5]. However, recent studies suggest that TLR10 has an important immunomodulatory role in macrophage responses to *S. pneumoniae* [7]. The signaling of TLR10 via the MyD88 or PIK3K pathway has been shown to reduce the production of IL-1β, while increasing the production of IL-1Ra [8]. TLR2 recognizes lipoteichoic acid, in the cell wall of *S. pneumoniae*, and also interacts with the protein porin B on the outer membrane of *N. meningitidis*. The activation of TLR2 triggers intracellular pathways that lead to the production of inflammatory cytokines. Along with TLR2, both TLR4 and TLR9 are involved in the inflammatory response to bacteria [9]. Studies on the pathogenesis of pneumococcal meningitis in mice have shown that the blockade of TLR2 and/or TLR2/4 signaling results in weakened host bacterial clearance, poor clinical signs, and more severe neurological complications [10]. We have previously shown that Angolan children with BM caused by *S. pneumoniae* who carried the variants of *TLR10* SNPs exhibited an increased risk of coexisting pneumonia [11].

It is well established that *H. influenzae* is primarily recognized by TLR4, which detects its lipooligosaccharide (LOS) [12]. However, studies have shown that specific structural modifications in the LOS of *H. influenzae* can alter its immunostimulatory properties and promote signaling through alternative pattern recognition receptors, like TLR2 [13].

In this study, we evaluated a newly identified 23 bp insertion/deletion polymorphism in the promoter region of *TLR2* (rs111200466) and its possible risk-enhancing role in a cohort of patients with BM. We also analyzed this variant, together with *TLR10* SNPs, in relation to its effects on susceptibility to, severity of, and outcomes from BM in Angolan children.

## 2. Materials and Methods

### 2.1. Study Design and Aim

This study cohort comprises 190 children diagnosed with BM who participated in two prospective, randomized clinical trials conducted at Luanda Children’s Hospital (Hospital Pediátrico David Bernardino), Angola. The patients were enrolled from 2005 to 2008 and from 2012 to 2017. Both studies, conducted by the same research group and using identical diagnostic criteria, examined the role of continuous β-lactam infusion combined with oral acetaminophen in BM. In the present study, only patients with an identified causative agent were included. Angola introduced routine immunization against *H. influenzae* type b in 2006 and the 13-valent pneumococcal conjugate vaccine in 2013. Consequently, during the second study period, *H. influenzae* meningitis became rare, and the number of pneumococcal meningitis cases also declined compared with the first period. The control group included 268 Angolan children who had no previous episodes of bacterial meningitis and showed no evidence of active infection at the time of sampling [14], who were enrolled in 2008 and 2017 [15]. The studies were approved by the Luanda Children’s Hospital Ethics Committee (ISRCTN62824827, NCT 01540838). BM patients were included in the study after their guardians provided written informed consent, either by signature or, in cases of illiteracy, by fingerprint [15,16]. Oral informed consent was also obtained from the guardians of controls. The subjects have been described in detail elsewhere [17].

### 2.2. Laboratory Tests, Clinical Findings, and Severity Factors of BM

The diagnosis was set by the attending physician, and the diagnostic criteria have been previously described [15,16]. Blood and cerebrospinal fluid (CSF) samples were collected as described earlier [15,16].

The CSF specimens were analyzed by microscopy to detect cell counts and the presence of bacteria. Moreover, the concentrations of glucose, protein, and matrix metalloproteinase-8 (MMP-8) were analyzed. CSF was cultured on blood and chocolate agar plates, and bacterial isolates were identified using routine microbiological procedures included Gram staining, evalution of colony morphology, and biochemical identification tests [15,16]. Beginning in October 2016, CSF samples were also sent to the National Institute for Communicable Diseases in Johannesburg, South Africa, for PCR-based identification of *N. meningitidis*, *S. pneumoniae*, and *H. influenzae* [16]. The detailed information on the laboratory tests and the identification of bacteria have been previously published [15,16].

The prognostic factors for poor outcome in BM have been previously identified by Pelkonen et al. [18]. In this study, the genotype distributions of *TLR2* SNP and *TLR2-TLR10* haplotype were compared with these previously identified factors, which can be divided into laboratory variables and clinical features. Analyzed laboratory variables were glucose, protein, leukocyte, and MMP-8 concentrations in CSF at admission. In addition, C-reactive protein (CRP) concentration and leukocyte count in blood were analyzed. The assessed clinical features were poor general condition, level of consciousness, convulsions, and Glasgow and Blantyre coma scores at admission. In addition, dyspnea, other infectious foci identified at admission and during hospital stay, neurological sequelae (other than ataxia), deafness, blindness, and fatal outcome were analyzed.

### 2.3. DNA Isolation and Genotyping

The samples for genetic studies were collected by a dried blood spot (DBP) collection card (PerkinElmer sample Collection Device, PerkinElmer, Waltham, MA, USA) and SK-1S DNA buccal swabs (Isohelix, Harrietsham, Kent, UK). The DNA from samples was extracted with the QIAamp^®^ mini-DNA extraction kit (Qiagen, Hilden, Germany) [15].

Primers for *TLR2* SNP rs111200466 are 5′-CACGGAGGCAGCGAGAAA-3′ and 5′-CTGGGCCGTGCAAAGAAG -3′ [19]. The PCR conditions are according to the enzyme of Invitrogen Platinum Taq DNA Polymerase (Thermo Fisher, Waltham, MA, USA). The analyses annealing temperature is 58 °C. PCR product size of ins/ins is 286 bp (one band) and del/del 264 bp (one band). The ins/del has two band sizes of 264 bp and 286 bp [20]. The running parameters for gel electrophoresis are 110 volt for 1 h and then 130 volt for 30 min [21]. The detection of *TLR10* SNPs by Sanger sequencing has been described elsewhere [11].

All methods described above were performed in accordance with the relevant guidelines and regulations.

### 2.4. Statistical Analyses

Statistical analyses were performed using the JMP Pro software for Windows, version 14 (SAS Camous Drive, Cary, NC, USA). Categorical variables were described by numbers (*n*) and proportions (%) and calculated using bivariate analysis with Fisher’s exact test. Continuous data were described by means and 95% confidence intervals (CIs) or median and IQR, as appropriate. Non-normally distributed data were compared with the Mann–Whitney U test, and a mixed linear model was used for repeated measurements. For haplotype analyses, Bonferroni correction was also performed. A *p*-value of less than 0.05 was considered significant. Multivariate analyses were not performed, and results are therefore based on unadjusted comparisons. The Hardy–Weinberg (HWE) test was used to calculate the observed genotype distribution in both the study cohort and the control group. Differences in the numbers of patients are due to missing or non-determined data.

### 2.5. Ethics Approval and Consent to Participate

The Luanda Children’s Hospital’s Ethics Committee approved the studies on 22 June 2005, and the amendments on 21 December 2007 and 19 January 2012. The trials were registered internationally (International Standard Randomized Controlled Trial Number ISRCTN62824827, 22 August 2005, and ClinicalTrials.gov identifier NCT 01540838, 29 February 2012). The patients were enrolled after written informed consent or, in cases of illiteracy, a fingerprint was obtained from the guardian. Written informed consent was obtained from healthy control children and/or their guardians. All procedures were performed in accordance with the Declaration of Helsinki.

## 3. Results

The main causative agents identified in the 190 BM cases were *S. pneumoniae* (*n* = 108), *N. meningitidis* (*n* = 43), and *H. influenzae* (*n* = 39). In this cohort, only a single causative agent was identified in each case, with no evidence of multi-bacterial infections. The median age of the children was 15 (range 1–147) months. Of the children, 92 (48.2%) were female and 98 (51.6%) male. Detailed patient data are presented in Table 1. The control group had a median age of 66 months (range 0–186 months). Gender was recorded for 191 participants: 37% (*n* = 71) were female and 63% (*n* = 120) were male.

*TLR2* polymorphism rs111200466 (del −196 to −174) was analyzed, and the frequencies of the polymorphisms in both the patient and control groups are presented in Table 2. In patients, particularly in those with meningitis caused by *H. influenzae*, the presence of *TLR2* deletion (ins/del or del/del) seemed to be more frequent than in the control group. However, the difference did not reach statistical significance (Table 2).

Next, the association between coexisting infections and *TLR2* polymorphism was evaluated in patients with pneumococcal meningitis. At the time of hospital admission, 34 patients presented with coexisting infections, including bronchopneumonia (*n* = 23, 67.6%), pneumonia (*n* = 5, 14.7%), otitis (*n* = 1, 2.9%), abscess (*n* = 1, 2.3%), and other infection (*n* = 4, 11.8%). During hospitalization, the coexisting infection rate increased to 56.5% (*n* = 61), including bronchopneumonia (*n* = 41, 67.2%), pneumonia (*n* = 6, 9.8%), otitis (*n* = 6, 9.8%), cellulitis (*n* = 2, 3.3%), abscess (*n* = 1, 1.6%), osteomyelitis (*n* = 1, 1.6%), or other infection (*n* = 4, 6.6%). The patients who carried the ins/del or del/del variant of *TLR2* rs111200466 were found to have a lower risk of coexisting infection (OR 0.32; 95% CI 0.14–0.71; *p* = 0.006). The reduced risk was most noticeable for coexisting bronchopneumonia and pneumonia (OR 0.23; 95% CI 0.095–0.54; *p* = 0.001) (Table 3).

No associations were observed between *TLR2* rs111200466 polymorphisms and the other studied factors, which are presented in Table 3.

Two *TLR10* SNPs (rs10856837 and rs11096956) were analyzed previously [11], and here, the haplotype analyses between *TLR2* and *TLR10* polymorphisms were performed. As presented in Table 4, the pneumococcal BM patients who carried the *TLR2-TL10* haplotype ins/del or del/del-CC-CC had a reduced risk of coexisting pneumonia (OR 0.2; 95% CI 0.06–0.6; *p* = 0.007, corrected *p* = 0.058).

## 4. Discussion

In this study, a newly identified 23 bp insertion/deletion polymorphism within the promoter region of *TLR2* (rs111200466) was evaluated among Angolan children with BM and controls. The minor allele frequency (MAF) of the deletion was 0.254 among the controls, almost identical to the African population (0.257) included in the 1000 Genomes Project [22]. *TLR2* is in the same gene cluster with *TLR1*, *TLR6*, and TLR10 at the 4p14 of chromosome four [8]. *TLR2* polymorphism rs111200466 is a 23 bp nucleotide deletion (in/del) from position −196 to −174 in the untranslated 5′-region. Thus far, little is known about the functional role of *TLR2* polymorphism. However, one study reported that the del/del genotype was more prevalent in patients with hepatocellular carcinoma than in healthy controls [23]. There is also evidence that the polymorphism of *TLR2* rs111200466 influences the expression of TLR2 [23,24,25] and the reduced pam3cys-inducible TLR2 expression on macrophages [26]. We have recently shown that healthy Finnish children who carry the ins/del or del/del genotype of *TLR2* rs111200466 had significantly lower levels of serum IL-33 and IL-17A than children with the ins/ins genotype [15]. In contrast, another study reported no association between *TLR2* rs111200466 and rheumatoid arthritis [27].

Although the precise function of the studied deletion remains unclear, it is well established that TLR2 plays a critical role in host defense against bacterial meningitis. Böhland et al. found that TLR2-deficient mice had a significantly increased mortality rate and higher bacterial burden in pneumococcal meningitis [28]. Previous studies have also shown the importance of other *TLR2* SNPs in meningitis. For example, one study conducted in Chinese children found that BM patients with variant CC of *TLR2* of SNP rs3804099 had a fourfold increased risk of having lower concentrations of glucose in CSF, and the patients with variant TC had an increased risk of seizures [27]. In a Dutch study, van Well et al. reported that patients with homozygous variant alleles of *TLR2* +2477 (rs5743708) (AA) and *TLR4* +896 (rs4986790) (GG) had an enhanced probability of developing meningococcal meningitis [29].

Although TLR10 belongs to the same subfamily with TLR2, unlike TLR2, the TLR10 ligand is unknown. However, recent studies suggest that TLR10 has an important immunomodulatory role in macrophage response to *S. pneumoniae* [7]. TLR10 through the MyD88 or PIK3K pathways has been suggested to lead to a reduction in IL-1β production and an increase in IL-1Ra production [30]. In addition, recent studies in murine models have reported that IL-1β has a major role in resistance to primary pneumococcal infection, whereas IL-1α appears to be less important [31].

We have previously shown with the same cohort that children who carry *TLR10* SNPs (rs10004195, rs10856837, or rs11096956) exhibit an increased risk of coexisting pneumonia [11]. Here, we observed an association of *TLR2* polymorphism rs111200466 with childhood meningitis with the three most common causative agents. The *TLR2* deletion was slightly more in the patient group than in controls. This was observed especially in patients with *H. influenzae*, which caused meningitis; however, differences were not statistically significant. Concomitant pneumonia is relatively common with non-meningococcal meningitis and has been associated with more severe disease and an increased risk for long-term complications. Therefore, we performed further analyses with pneumococcal meningitis patients. We found that patients who carried the ins/del or del/del variant of *TLR2* rs111200466 had a lower risk of coexisting infection (OR 0.32; 95% CI 0.14–0.71; *p* = 0.006). The reduced risk was most noticeable for coexisting bronchopneumonia and pneumonia (OR 0.23; 95% CI 0.095–0.54; *p* = 0.001). The deletion thus seems to lower the risk of coexisting infection, especially pneumonia. We also performed haplotype analyses between *TLR2* SNP and previously detected *TLR10* SNPs. It seems that a variant genotype of *TLR2* rs111200466 together with a wildtype of *TLR10* SNPs (rs10856837 and rs11096956) may protect against coexisting pneumonia [11]. It should be kept in mind that we did not find any patient in this cohort with ins/del or del/del of *TLR2* rs111200466 who carried either variant type *TLR10* rs10856837 or TLR10 rs11096956 (Table 4), suggesting that these genotype combinations may be rare and deleterious in this population. The statistical power for the subgroup and haplotype analyses was limited, which reduces the strength of the inferences that can be drawn from these results.

Overall, approximately 43% of patients with community-acquired bacterial meningitis have coexisting infections, with the highest rate observed in pneumococcal meningitis (51%). These rates vary depending on age, underlying conditions, and the causative pathogens [28]. A recent study by Pelkonen et al. showed a relationship between coexisting pneumonia and young age and lower living standards [3]. In addition, among the pathogens that cause BM, *S. pneumoniae* has the highest rate (18–22%) of associated pneumonia, and BM with coexisting pneumonia is associated with poor outcome and death [3].

We acknowledge some limitations in our study. The total number of children with confirmed BM—and particularly those with specific causative pathogens—was limited, which may have resulted in missed associations. Additionally, we examined only one SNP in *TLR2* and two SNPs in *TLR10*; the potential influence of other SNPs on susceptibility to and outcomes of BM should also be considered. Missing results were infrequent and occurred randomly; therefore, they did not introduce systematic selection bias.

## 5. Conclusions

Our study suggests that there is an association between the polymorphism of *TLR2* rs111200466 and coexisting pneumonia in patients with pneumococcal meningitis. Furthermore, patients with pneumococcal meningitis who carry a variant genotype (ins/del or del/del) of *TLR2* rs111200466 seem to have a reduced risk of pneumonia. The results of this study are valid in the Angolan population, and further research is warranted to explore the impact of *TLR2* polymorphism on the outcome of BM in other ethnic populations.

## Figures and Tables

**Table 1:** Demographic, clinical, and laboratory information on patients with *Streptococcus pneumoniae*, *Haemophilus influenzae*, and *Neisseria menigitidis* infection.

Characteristics	Total Subjects ^1^	*n* (%)	Value Distribution	Range
Age in months, median (IQR)	190	-	15 (Q1: 6.8; Q3: 44.3)	1–147
Sex, male	190	98 (51.6%)	-	-
Weight (kg), median (IQR)	189	-	9.2 (Q1: 6.9; Q3: 12.8)	4.5–36
Clinical features				
Fever (°C), mean (SD)	187	70 (37.4%) ^2^	37.5 (0.96)	35.3–40.0
Poor general condition	187	113 (60.4%)	-	-
Convulsions in hospital	185	91 (49.2%)	-	-
Altered consciousness at admission	187	133 (71.1%)	-	-
Glasgow Coma Score, median (IQR)	168	-	12 (Q1: 9; Q3: 15)	3–15
Dyspnea	188	98 (52.1%)	-	-
Coexisting infection at admission	184	55 (29.9%)	-	-
Pneumonia at admission	184	43 (23.4%)	-	-
Coexisting infection during hospitalization	190	105 (55.3%)	-	-
Pneumonia during hospitalization ^4^	190	76 (40.0%)	-	-
Fatal outcome	190	47 (24.7%)	-	-
Length of hospital stay in days, median (IQR)	128 ^3^	-	12 (Q1: 9; Q3: 17)	6–38
Severe neurological sequelae at discharge	142	19 (13.4%)	-	-
Deaf	116	15 (12.9%)	-	-
Blind	139	13 (9.4%)	-	-
Neurological sequelae without ataxia	136	29 (21.3%)	-	-
Ataxia	134	41 (30.6%)	-	-
Laboratory tests, median (IQR)				
CRP (mg/L)	159	-	161 (Q1: 128; Q3: 161)	7–239
Glucose in CSF (mg/dL) ^5^	182	-	9.9 (Q1: 5.4; Q3: 18.5)	0.5–270
Protein in CSF (mg/dL) ^5^	88	-	204.3 (Q1: 144.9; Q3: 268.2)	10.2–1404
Leukocytes in CSF (/mm^3^) ^5^	189	-	1600 (Q1: 560; Q3: 3640)	10–32,400
Blood leukocytes (/µL)	140	-	15.6 (Q1: 10.7; Q3: 21.8)	1.23–50.7
MMP-8 in CSF (ng/mL)	75	-	766.4 (Q1: 258.7; Q3: 1214.9)	16.7–2390.3

^1^ Total number of subjects in dataset is 190; numbers less than 190 indicate those for whom information was available. ^2^ Axillary temperature, fever if ≥38.0 °C. ^3^ Length of hospital stay excludes patients with fatal outcome. ^4^ Pneumonia (*n* = 15) and bronchopneumonia (*n* = 61). ^5^ Measured at admission. - = not applicable or not reported.

**Table 2:** Genotype frequencies of analyzed *TLR2* rs111200466 in patients with bacterial meningitis caused by *H. influenzae*, *N. Meningitidis*, or *S. pneumoniae*.

	Patients (All)	*H. influenzae*	*N. meningitidis*	*S. pneumoniae*	Control Group
ins/ins (%)	95 (50.3)	16 (41.0)	26 (60.5)	53 (49.5)	157 (58.6)
ins/del (%)	77 (40.7)	20 (51.3)	12 (27.9)	45 (42.1)	86 (32.1)
del/del (%)	17 (9.0)	3 (7.7)	5 (11.6)	9 (8.4)	25 (9.3)
ins (%)	267 (70.6)	52 (66.7)	64 (74.4)	151 (70.6)	400 (74.6)
del (%)	111 (29.4)	26 (33.3)	22 (25.6)	63 (29.4)	136 (25.4)
Total *n*	189 ^3^	39	43	107	268
HWE		0.34	0.08	0.90	0.01
*p*-value	0.079	0.056	0.869	0.134	Reference *n*
OR (95% CI) ^1^	1.4 (1.0–2.0)	2.0 (1.0–4.0)	0.9 (0.5–1.8)	1.4 (0.9–2.3)	
*p*-value	0.181	0.138	0.967	0.255	Reference *n*
OR (95% Cl) ^2^	1.2 (0.9–1.6)	1.48 (0.9)	1.0 (0.6–1.7)	1.2 (0.9–1.7)	

^1^ All genotypes compared with controls (Reference *n*). ^2^ Ins—del allele ratios compared with controls (ref.). ^3^ One patient did not have sufficient DNA available for the TLR2 deletion analysis.

**Table 3:** Associations between *TLR2* rs111200466 polymorphism and laboratory values and severity factors of *S. pneumoniae*-caused bacterial meningitis. Q25% Q75%.

Clinical Features	rs111200466		OR (95% Cl)	*p*-Value
	ins/ins	ins/del and del/del		
Poor general condition ^1^	39 (73.6)	35 (67.3)	0.74 (0.32–1.72)	0.526
Convulsions at admission ^1^	35 (66.0)	26 (50.0)	0.51 (0.23–1.13)	0.115
Level of consciousness ^1^				
Normal	10 (19.2)	8 (14.8)	-	0.670
Altered	33 (63.5)	39 (72.2)	-	
Coma	9 (17.3)	7 (13.0)	-	
Glasgow Coma Score (≤12) ^1^	30 (69.8)	31 (67.4)	0.90 (0.37–2.19)	0.824
Dyspnea	35 (66.04)	33 (62.26)	0.84 (0.38–1.88)	0.840
Other focus of infection at admission	24 (46.2)	10 (18.9)	0.27 (0.11–0.65)	0.004
Pneumonia at admission	22 (44.0)	6 (12.24)	0.18 (0.06–0.49)	0.001
Other focus of infection during hospital stay	37 (69.8)	23 (42.6)	0.32 (0.14–0.71)	0.006
Pneumonia during hospital stay	32 (66.7)	14 (31.1)	0.23 (0.09–0.54)	0.001
Outcome at discharge				
Fatal	17 (32.1)	20 (37.0)	1.25 (0.56–2.77)	0.685
Severe neurological sequelae	7 (19.4)	6 (17.7)	0.89 (0.27–2.97)	1.000
Deafness	4 (14.3)	6 (22.2)	1.71 (0.43–6.91)	0.503
Blindness	5 (13.9)	4 (12.1)	0.86 (0.21–3.50)	1.000
Any neurological sequelae (no ataxia)	11 (32.4)	9 (28.1)	0.82 (0.29–2.35)	0.792
Ataxia	12 (35.4)	11 (35.5)	1.00 (0.36–2.79)	1.000
Laboratory variables (IQR)				
CRP (mg/L)	161 (118.0; 161.0) (*n* = 43)	161 (135.3; 179.8) (*n* = 48)	-	0.090
CSF-glucose (mg/dL) ^1^	8.6 (6.3; 15.5) (*n* = 51)	10.0 (4.8; 12.6) (*n* = 54)	-	0.266
CSF-protein (mg/dL) ^1^	232.5 (167.0; 293.4) (*n* = 26)	202.3 (164.3; 259.4) (*n* = 28)	-	0.291
CSF-leukocytes (/mm^3^) ^1^	1500 (355.0; 3790) (*n* = 53)	1420 (293.8; 2850) (*n* = 54)	-	0.825
Blood leukocytes (/µL)	15.3 (9.5; 23.7) (*n* = 37)	15.4 (10.4; 19.3) (*n* = 33)	-	0.773
CSF-MMP-8 (ng/mL) median	627.6 (163.6; 922.0) (*n* = 22)	494.4 (157.9; 874.3) (*n* = 18)	-	0.606

^1^ Determined/measured at admission.

**Table 4:** Haplotype analysis of one *TLR2* and two *TLR10* SNPs in patients with pneumococcal meningitis, comparing patients with and without coexisting pneumonia.

TLR2: rs111200466	TLR10: rs10856837	TLR10: rs11096956	Pneumonia *n* = 46 (%)	No Coexisting Infections *n* = 47 (%)	*p*-Value ^1^	OR (95% CI)
Wildtype	Wildtype	Wildtype	17 (28.8)	13 (27.7)	Reference *n*	Reference *n*
Wildtype	Wildtype	Variant	1 (1.7)	0	1	-
Wildtype	Variant	Wildtype	0	0	1	-
Wildtype	Variant	Variant	14 (23.7)	3 (6.4)	0.11	3.6 (0.8–15.1)
Variant	Wildtype	Wildtype	6 (10.2)	23 (48.9)	0.007 ^2^	0.2 (0.06–0.6)
Variant	Wildtype	Variant	0	0	1	-
Variant	Variant	Wildtype	0	0	1	-
Variant	Variant	Variant	8 (13.6)	8 (17.0)	0.76	0.8 (0.2–2.6)

For TLR2, wildtype indicates ins/ins, and variant indicates ins/del or del/del. For TLR10, rs10856837 wildtype indicates CC and variant indicates CT or TT genotypes, rs11096956 wildtype indicates CC and variant AC or AA genotypes. ^1^ *p*-value was calculated by comparing with wildtype–wildtype–wildtype. ^2^ *p* = 0.058 after Bonferroni correction (multiplying the *p*-value by the number of paired comparisons).

## Data Availability

The datasets used and/or analyzed during the current study are available from the corresponding author on reasonable request.

## Abbreviations

The following abbreviations are used in this manuscript:

BM	Bacterial meningitis
bp	Base pair
CFS	Cerebrospinal fluid
Cl	Confidence interval
CRP	C-reactive protein
DBP	Dried blood spot
HWE	Hardy–Weinberg equilibrium
IL	Interleukin
MMP8	Matrix metalloproteinase-8
OR	Odds ratio
PCR	Polymerase chain reaction
PRRs	Pattern recognition receptors
SNP	Single nucleotide polymorphism
TLR	Toll-like receptor
